# Osteonecrosis of the Jaw

**DOI:** 10.3390/dj11010023

**Published:** 2023-01-09

**Authors:** Božana Lončar Brzak, Lorena Horvat Aleksijević, Ema Vindiš, Iva Kordić, Marko Granić, Danica Vidović Juras, Ana Andabak Rogulj

**Affiliations:** 1 Department of Oral Medicine, School of Dental Medicine, University of Zagreb, 10000 Zagreb, Croatia; 2 Faculty of Dental Medicine and Health, University of Osijek, 31000 Osijek, Croatia; 3 Dental Practice at Healthcare Center Ormož, 2270 Ormož, Slovenia; 4 Independent Researcher, 10000 Zagreb, Croatia; 5 Department of Oral Surgery, School of Dental Medicine, University of Zagreb, 10000 Zagreb, Croatia; 6 Department of Oral Medicine, University Clinical Hospital Zagreb, 10000 Zagreb, Croatia

**Keywords:** drug-induced osteonecrosis, osteonecrosis, osteoradionecrosis, radiotherapy

## Abstract

Osteonecrosis of the jaw is a condition in which bone cells die due to various causes. It is classified as drug-induced jaw osteonecrosis, osteoradionecrosis, traumatic, non-traumatic, and spontaneous osteonecrosis. Antiresorptive or antiangiogenic drugs cause drug-induced osteonecrosis. The combination of medications, microbial contamination, and local trauma induces this condition. Osteoradionecrosis is a severe radiation therapy side effect that can affect people with head and neck cancer. It is described as an exposed bone area that does not heal for longer than three months after the end of radiation treatment with the absence of any indications of an original tumor, recurrence, or metastasis. Trauma (tooth extraction), tumor site, radiation dose that the patient receives, the area of the bone which is irradiated, oral hygiene, and other factors are risk factors for the development of osteonecrosis. Less frequently, osteonecrosis can also be induced by non-traumatic and traumatic causes. Non-traumatic osteonecrosis is brought on by infections, acquired and congenital disorders, as well as the impact of chemicals. Traumatic osteonecrosis is brought on by thermal, mechanical, or chemical damage. The treatment of osteonecrosis can be conservative, which aims to be beneficial for the patient’s quality of life, and surgical, which involves debridement of the necrotic bone.

## 1. Introduction

Osteonecrosis refers to several conditions that lead to bone damage and its disruption. The causes of osteonecrosis can be different and occur in different bones in the body. Avascular osteonecrosis is associated with partial or complete loss of blood supply and most often occurs in the femur [1]. A new type of osteonecrosis was described in 2003, and it refers to necrosis of the jaw bone associated with the use of bisphosphonate drugs. Other types of drugs can also cause osteonecrosis besides bisphosphonates [2]. Osteoradionecrosis is a term that refers to bone necrosis caused by radiation therapy. Radiation leads to inflammation and obliteration of the blood vessels supplying the bone, causing avascular necrosis with hypoxic, hypovascular, and hypocellular lesions [3]. Traumatic osteonecrosis is caused by physical, chemical, or thermal bone trauma. Non-traumatic osteonecrosis is associated with infections, neoplasms, use of narcotics, and vascular causes such as ischemia, occlusion, coagulopathy, hemoglobinopathy, and some autoimmune diseases. However, some cases of idiopathic osteonecrosis that developed without an obvious etiological cause have also been described [4,5].

The purpose of this paper is to present different types of osteonecrosis of the jaw, the risk factors for their occurrence, treatment options, and prevention.

## 2. Types of Osteonecrosis

Osteonecrosis occurring in the jaw can be divided into medication-related osteonecrosis of the jaw (MRONJ), osteoradionecrosis (ORN), traumatic osteonecrosis, non-traumatic osteonecrosis and spontaneous osteonecrosis (Table 1).

### 2.1. Medication Related Osteonecrosis of the Jaw

Medication related osteonecrosis of the jaw significantly affects the patient’s quality of life and leads to a significant percentage of mortality. This condition is associated with problems in swallowing, feeding, chewing, and speaking, as well as the appearance of swollen and painful mucous membranes, chronic sinusitis, and others [6].

The main criteria for the diagnosis of this type of osteonecrosis, which is generally used, are:

- current or previous therapy with antiresorptive or antiangiogenic drugs
- exposed or necrotic bone in the maxillofacial region that persists for more than eight weeks
- the patient was not irradiated in the jaw area.

However, in the workshop of the European task force on MRONJ, which was held in 2019, it was proposed that an 8-week observation period should not be a prerequisite for establishing the diagnosis of MRONJ [7]. A newer definition says that MRONJ is an “adverse drug reaction described as the progressive destruction and death of bone that affects the mandible and maxilla of patients exposed to the treatment with medications known to increase the risk of disease, in the absence of a previous radiation treatment” [8]. 

Diagnostic criteria are important since osteonecrosis can be differentially confused with other conditions, such as alveolar osteitis, sinusitis, gingivitis/periodontitis, periapical lesions, and disorders of the temporomandibular joint [6]. 

#### 2.1.1. Pathophysiology

The exact mechanism of its occurrence is still unknown. It is considered that the occurrence of MRONJ is a combination of the interaction of drugs with the presence of microbiological contamination of a certain area, as well as local trauma [9]. Antiresorptive and antiangiogenic drugs are mostly prescribed to stabilize the loss of bone mass caused by osteoporosis in women but also for the treatment of spreading lesions of bone cancer and other malignant conditions. The mechanism of osteoporosis treatment is the inhibition of trabecular bone resorption by osteoclasts [10,11]. Antiresorptive drugs inhibit the differentiation and function of osteoclasts, which leads to their apoptosis and causes a decrease in bone resorption and remodeling. In addition to the effect on osteoclasts, bisphosphonates negatively affect the biological activity of osteoblasts, fibroblasts, and keratinocytes. The death of fibroblasts is especially visible in the oral epithelial cells, which leads to bone exposure and impaired healing and plays a leading role in the development of osteonecrosis [9,11]. In the pathogenesis of osteonecrosis, a key role is also played by inflammation at the site of origin caused by bacterial contamination. Bisphosphonates increase the adhesion of bacteria to hydroxylapatite in the bone, which becomes necrotic and avascular, which is why the most common cause of osteonecrosis is a tooth extraction or advanced periodontal disease [12], while in 30% of cases, MRONJ develops spontaneously [11]. All medications that cause osteonecrosis also lead to reduced angiogenesis, which leads to ischemia. One of the most important factors for the formation of MRONJ is the pH value. When pH values are low, bisphosphonates are released from bone and activated to bind to osteoclasts and inhibit their activity, affecting osteoblasts, fibroblasts, macrophages, and lymphocytes [13]. The therapeutic indication for which the medication is prescribed is among the most important parameters for risk estimation for the development of MRONJ. Cancer patients who take oral antiresorptive medications have a greater risk for the development of MRONJ than osteoporotic or osteopenic patients [14]. Zolendronate and denosumab are the drugs that are most frequently associated with MRONJ [15]. Cancer patients who are treated with zolendronate have a cumulative risk of MRONJ lower than 5%, while in patients treated with denosumab, the risk is similar, less than 5%, with a range of 0–6.9% [14]. Osteoporotic patients treated with oral bisphosphonates have a risk of MRONJ development of 0.02–0.05%, and for IV zolendronate, the risk is ≤0.05%. In osteoporotic patients treated with denosumab, the risk is 0.3%. Longer duration of antiresorptive therapy is also among the known risk factors. Among local factors, the most important one are dentoalveolar procedures. In osteoporotic patients on bisphosphonates, the risk for MRONJ after a tooth extraction is 0–0.15%, and for patients on denosumab, the risk is 1%. For cancer patients on bisphosphonates, the risk for MRONJ after tooth extraction varies from 1.6–14.8% [14]. Other local factors, demographic and systemic factors and other medications should also be considered when estimating the risk for MRONJ development [6,14]. 

#### 2.1.2. Clinical Status

The main clinical indicator of MRONJ is exposed bone which can vary from small exposed edges of an empty alveolus to the entire jaw or both jaws [16]. Along with the exposed lesion, there are often signs of inflammation, such as increased volume of soft tissues, with or without suppuration, limited purulent inflammation, or fistula. The mandible is most often affected due to poor blood supply and thin mucosa, compared to the upper jaw [17]. Symptoms of osteonecrosis depend on the course of the disease and its spread to the surrounding structures. Pain occurs in the acute stage, and after the onset of necrosis, it is asymptomatic. Further progression can cause numbness, oroantral communication, and pathological fracture of the jaw [6]. Radiological analysis is used for monitoring the course of the disease, involvement of the region, and complications of osteonecrosis. The most common visible change is the sclerosis of the lamina dura of the alveolar bone. Changes in the trabecular pattern or unexplained bone resorption, as well as persisting alveolar sockets after tooth extraction, can also be seen [2,6]. In patients with bone exposure, focal and diffuse bone sclerosing and sequestration can be seen more frequently than in those without bone exposure [18]. In the late stages of separating necrotic bone, a detached sequestrum with marked margins can be present [19]. Computed tomography (CT) or cone beam computed tomography (CBCT) gives a detailed view of a lesion progression. Magnetic resonance imaging (MRI) and scintigraphy may also be used [8,13,20].

#### 2.1.3. Classification

Stage 0 comprises patients in whom osteonecrosis is not clinically evident but has subjective complaints or radiological indicators that may be related to osteonecrosis. Symptoms that occur are odontalgia without the visible cause, dull pain in the mandible, and sinus pain [6,11], and the diagnosis is challenging. Clinical manifestations include unexplained tooth loss, fistula not associated with pulp necrosis or caries, and gingival swelling. Radiological signs include resorption of alveolar bone that cannot be explained by chronic periodontitis, changes in trabecular pattern, impaired wound healing, and alveolar or surrounding bone sclerosing [21,22,23]. The therapy of this stage is conservative and symptomatic, as well as patient education [6].

Stage 1 includes clinically exposed dead bone or fistula arising from bone, with no signs of infection and no symptoms. The treatment consists of follow-up of the lesion and removal of the bone in case of bone sequestrations [6,11].

**Figure 1:**
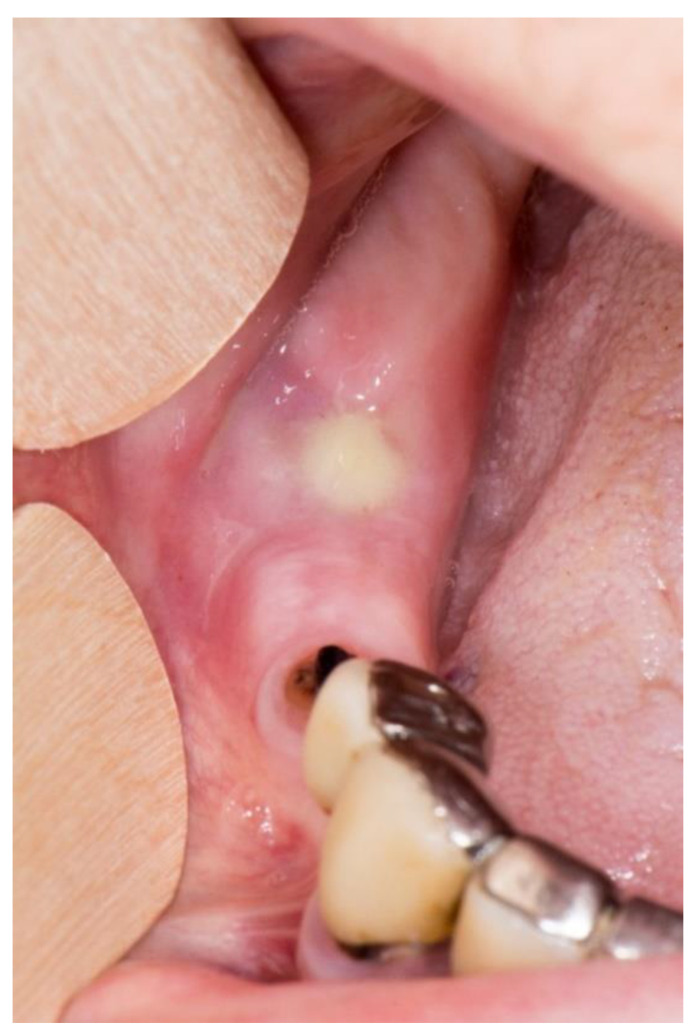
Suppuration in the area of necrotic bone in zolendronate-related osteonecrosis of the jaw.

**Figure 2:**
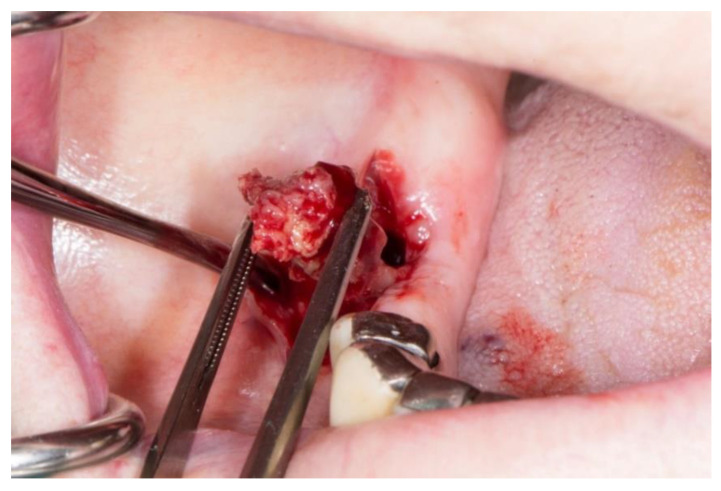
Surgical removal of necrotic bone fragment.

**Figure 3:**
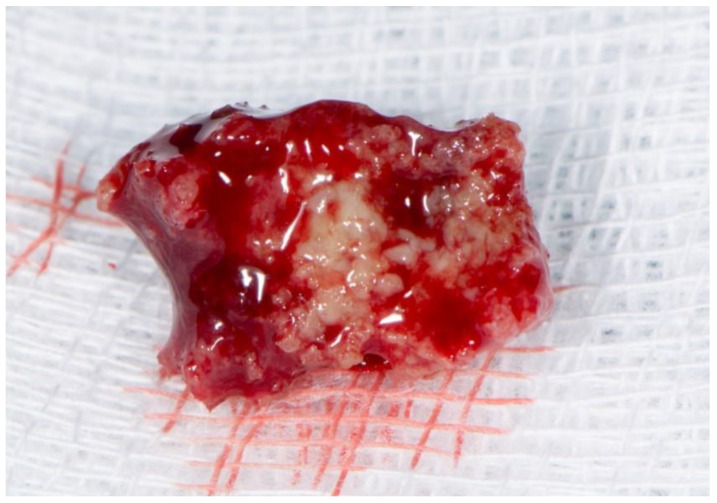
Necrotic bone fragment.

**Figure 4:**
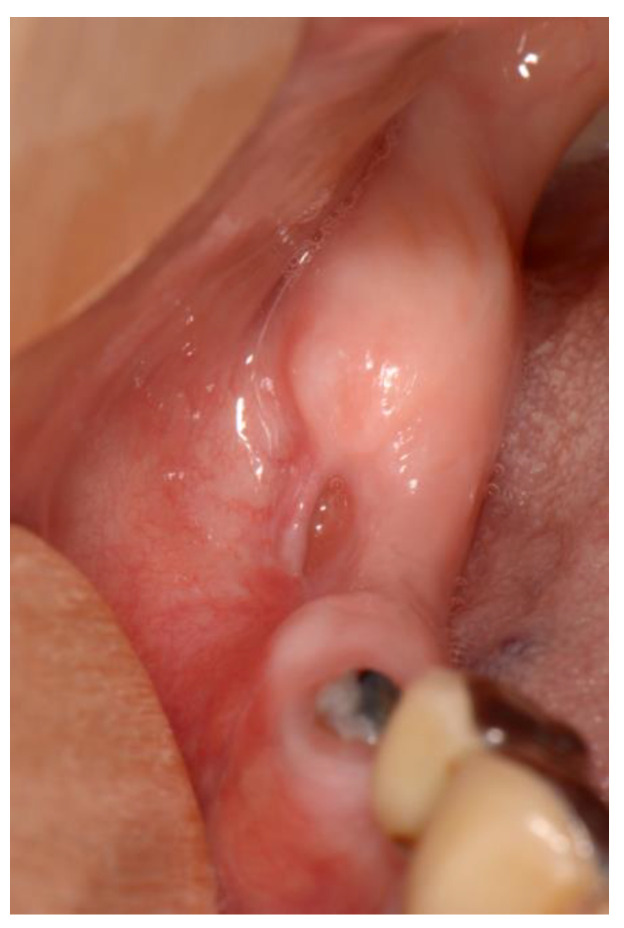
Healing after surgical removal of necrotic bone and sanitizing defect with the use of plasma rich in growth factors (PRGF)-Endoret technology.

Stage 2 includes patients with necrotic bone, which is visible during an examination, or with the presence of a fistula and complaints of painful symptoms (Figure 1). The therapy of this stage is aimed at healing, promoting antibiotic therapy, and antisepsis with chlorhexidine. After the inflammation subsides, it is necessary to debride the area [6] (Figure 2, Figure 3 and Figure 4).

Stage 3 includes clinically visible necrotic bone or a fistula with signs of acute infection. The patient complaints of pain and has one of the following signs: death of tissue outside the alveoli, extraoral fistula, progressive destruction of the lower border of the lower jaw and the lower part of the maxillary sinus with the appearance of oroantral fistula and a tendency to pathological fractures. The therapy is surgical and antibiotic treatment, with the reconstruction of the surgically removed bone [6,11].

#### 2.1.4. Prevention

A multidisciplinary approach to the treatment of patients is important for the prevention of medication-related osteonecrosis. Dental examination and necessary dental procedures before starting therapy significantly reduce the risk of medication-related osteonecrosis of the jaw. Treatment planning should include a thorough examination of the oral cavity and radiographic analysis. It is important to identify acute infections, as well as places of potential infection, and to rehabilitate them in time. All extractions of teeth with a poor prognosis should be performed at least three weeks before the therapy. It is necessary to educate patients about the risk of osteonecrosis and to motivate them to maintain oral hygiene and follow-up examinations. If the patient is already receiving therapy, it is sometimes necessary, in agreement with the responsible doctor, to remove the therapy for a certain period of time in order to achieve adequate dental treatment [6,8,11].

#### 2.1.5. Medication That Can Cause MRONJ

Medication that can cause MRONJ can be divided into antiresorptive drugs, which include bisphosphonates and denosumab, and antiangiogenic drugs [20].

Bisphosphonates are analogs of pyrophosphate, a natural inhibitor of bone metabolism. Their mechanism of action is the inhibition of osteoclasts, which leads to their apoptosis and suppresses bone remodeling. Bisphosphonates show a high affinity for the hydroxyapatite matrix of the bone in which they are incorporated, changing the bone microstructure, which slows down the growth and dissolution of minerals in the bone. Osteoblastic activity remains preserved, which results in an increase in bone mass [24]. The main side effect of bisphosphonates is osteonecrosis of the jaw, but other side effects can also occur, such as gastrointestinal disturbances, atypical femur fractures, inflammation of the esophagus with mucosal erosions, secondary hyperparathyroidism, atrial fibrillation, eye discharge, muscle pain, and other [25]. Bisphosphonates can be administered orally or parenterally.

Oral administration is indicated for osteoporosis, osteopenia, Paget’s disease, and osteogenesis imperfecta [26], as well as in the treatment of chronic kidney disease, kidney transplantation, rheumatoid diseases associated with systemic bone loss, and non-inflammatory rheumatoid diseases [27]. They are less potent for causing osteonecrosis compared to parenteral administration. Parenteral bisphosphonates are used to treat various conditions associated with malignant diseases. Parenterally applied bisphosphonates stimulate innate antitumor immune mechanisms and thus inhibit the growth and formation of bone metastases, most often in breast and prostate cancer [8,26].

Denosumab is a humanized monoclonal antibody targeting the modulation regulator (RANK ligand) that inhibits osteoclasts and reduces bone resorption [26] and is used in the treatment of osteoporosis and bone lesions in malignant diseases. It is applied subcutaneously and does not accumulate in the bone, and its impact on remodeling is reversible [8].

Antiangiogenic drugs prevent the formation of new blood vessels by binding to various signaling molecules that inhibit angiogenesis. These include bevacizumab and sunitinib. Bevacizumab is a humanized monoclonal antibody that selectively binds to human vascular endothelial growth factor (VEGF), which is found on the lining of blood and lymphatic vessels in the body. Bevacizumab is used to treat malignant diseases of the kidney, gastrointestinal tract, lung, and glioblastoma [27,28]. Sunitinib is a thyroxine kinase inhibitor used in treating gastrointestinal tumors, metastatic renal cell carcinomas, and pancreatic neuroendocrine tumors [29]. In combination with chemotherapy or with bisphosphonates, they have a high potency to cause osteonecrosis [28].

#### 2.1.6. Risk Assessment

Risk assessment for the development of MRONJ depends on the administration of the drug, duration of treatment, dosing, and potency, but also some local, anatomic and systemic factors. As previously mentioned, parenteral or subcutaneous administration is more potent for causing osteonecrosis than oral administration [1,8,26]. Greater doses and longer duration of the therapy also increase the risk. Invasive dental procedures such as tooth extraction, alveotomy, placement of dental implants, and endodontic and periodontal surgery increase the risk of developing osteonecrosis 5–7 times [1,8,26]. Poor oral hygiene, periodontitis, periapical inflammation, and other inflammatory oral conditions are also considered as risk factors [8]. MRONJ usually affects the mandible, especially the lingual side, which is covered with thin mucosa [1,8]. Systemic factors for developing osteonecrosis include age, gender, other systemic diseases, and medications. Osteonecrosis usually develops in older women [30]. Treatment with corticosteroids or chemotherapy and diseases such as rheumatoid arthritis, systemic lupus, hyperthyroidism, renal insufficiency, and smoking represent additional risks [8,11].

#### 2.1.7. Therapy

The therapy of osteonecrosis depends on the degree of development of the disease, and there are two different approaches to the treatment of MRONJ. The first approach prefers conservative before surgical therapy [1,11], while the other is the opposite [20]. Conservative therapy includes systemic antibiotic therapy in combination with antimicrobial therapy with chlorhexidine. Surgery is planned only if the disease progresses after failed conservative therapy. The second approach gives preference to surgical therapy because it is considered necessary to remove the necrotic part of the bone at all times since such bone cannot be revitalized and, as such, creates a nutrient base for the colonization of microorganisms and further progression of the disease. Some evidence from the literature point out the benefit of the surgical approach in treatment, even at an earlier stage MRONJ, but the decision should be made individually for each patient [16], taking into consideration the potential benefit of the surgery on the general health status of the patient [8]. The necrotic bone removed during surgery is recommended to be sent for histopathological processing [31]. Surgical techniques include sequestrectomy, ridge modeling, and jaw resection with different reconstructive methods. It has been proven that surgical interventions can be more successful in controlling the disease itself compared to a conservative approach [32]. Ablation of necrotic bone can be done conventionally or with Er-YAG lasers [33,34].

Treatment of MRONJ depends on bone and soft tissue repair. After removal of the necrotic part, the surrounding bone should be modeled, and the soft tissue primarily sutured without tension, although some surgeons recommend double covering of the exposed part of the bone with a muscle flap or a buccal fat tissue flap [11,35,36]. Positive results from the topical application of minocycline in orabase as adjuvant therapy after surgical debridement were published a few years ago, but the results should be confirmed in a larger number of patients [37]. Additional possible therapeutic options include hyperbaric oxygenation, ozone therapy, laser therapy, and the application of growth factors in combination with antibiotics to reduce the lesion and relieve symptoms [11,38,39]. Hyperbaric oxygenation is contraindicated in patients with malignant diseases because it increases circulation and can encourage the spread of disease [40]. Low-level laser treatment can be used for biostimulation alone or as a part of a combined approach [41,42].

Data from the literature show that a combination of laser ablation and LLLT is more successful in the treatment of MRONJ than LLLT alone [43].

Based on the literature results, vitamin D supplementation represents a low-risk and low-cost type of treatment and might also be useful for the prevention or treatment of MRONJ in patients with vitamin D deficiency. Vitamin D is important for bone mineralization, angiogenesis, and inflammatory response, which are all mechanisms included in the development of MRONJ [44]. It is shown that the active form of vitamin D decreases the number of osteoclasts and promotes bone production, regulates angiogenesis, and reduces inflammatory response [44]. Results from the literature regarding low levels of vitamin D in patients with jaw osteonecrosis are conflicting, with some studies indicating that low levels of vitamin D represent a risk factor for the development of osteonecrosis [45,46], while others deny it [47].

Based on the abovementioned results, supplementation of vitamin D in patients with a deficiency of vitamin D might be beneficial for the prevention or treatment of MRONJ, but future studies should define definitive clinical guidelines.

### 2.2. Osteoradionecrosis

Osteoradionecrosis of the jaw is a rare but extremely serious complication of radiation therapy in patients with head and neck cancer [3]. Osteoradionecrosis is defined as an area of exposed, irradiated bone that does not heal for more than three months, and there are no signs of a primary tumor, recurrence, or metastasis [48].

#### 2.2.1. Pathophysiology

Different mechanisms of pathogenesis were proposed. Marx suggested that radiation leads to radiation arteritis, which consequently induces the formation of hypoxic, hypovascular, and hypocellular tissue. Due to hypoxia, the tissue cannot be renewed, and the wounds cannot heal [49,50].

Another theory is radiation-induced fibrosis, which presumes that radiation induces changes in fibroblastic activity, which happen during three different phases: prefibrotic phase, continuously organized phase, and late fibroatrophic phase. Due to radiation and the formation of free radicals, endothelial cells are injured, after which released cytokines stimulate an inflammatory response. Different cytokines interfere with the healing process, leading to the formation of less valuable tissue that is susceptible to reactivated inflammation in case of local damage. The number of cells in the bone decreases, and the normal bone matrix is replaced by fibrotic tissue [51,52].

#### 2.2.2. Clinical Status

The diagnosis of osteoradionecrosis is established by clinical examination. Areas of exposed bone are visible in the oral cavity of patients who have been irradiated in the head and neck area. In addition to the necrotic bone, areas of ulceration and necrosis of the skin or mucosa are often visible [11,51]. Osteoradionecrosis is more common in the mandible, which has a thicker cortex and weaker blood supply than the maxilla. Patients may complain of pain, bad breath, dysgeusia, trismus, and difficulty chewing, swallowing, and speaking. The progression of osteoradionecrosis often leads to pathological fractures, extraoral or intraoral fistulas, and local or systemic infection [11,53]. Symptoms do not have to be present to diagnose osteoradionecrosis, especially in the early stages of the disease [51]. The surface of the wound may be infected by microorganisms in the mouth, but the deeper layers of the wound are not infected [49]. As initial radiographic diagnostic methods, plain radiographs and intraoral dental radiographs may be used, but CBCT and multidetector CT imaging is more precise in evaluating the extent of the lesions [52]. Magnetic resonance imaging (MRI) is more appropriate for soft tissue lesions near the mandible and be used in some patients. Positron emission tomography (PET) is also sometimes recommended for the differentiation of ORN from tumor recurrence, but it should be kept in mind that ORN-induced inflammation can give false positive results [52].

#### 2.2.3. Classification

According to the clinical course of the disease and response to therapy, several classifications of osteoradionecrosis can be found.

Morton and Simpson classify this type of osteonecrosis into three groups. In the first or mild form of the disease, it is manifested as ulceration and exposed bone, and the wound resolves spontaneously within a few months. The second group, also known as the moderate type, had bone exposure and isolation and improved after six months to one year of conservative treatment. The third group, i.e., the predominant form, is characterized by extensive bone exposure, the formation of larger possible fracture barriers, and the formation of fistulas. This form usually progresses rapidly, lasts more than a year, and requires surgical treatment [54].

**Figure 5:**
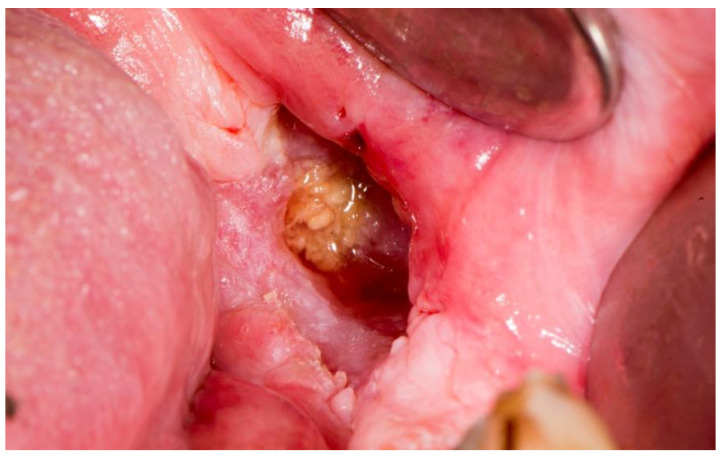
Necrotic bone in osteoradionecrosis.

**Figure 6:**
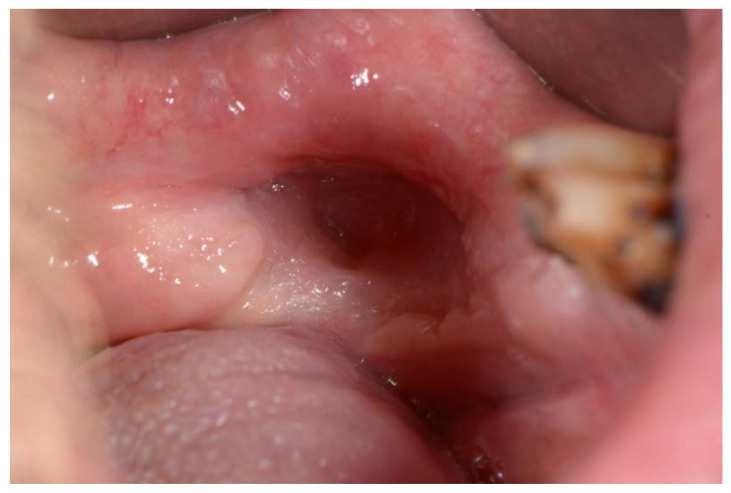
Healing of the bone and the mucosa after surgical intervention.

Kagan and Schwartz described a three-stage system based on clinical assessment and physical examination. The first stage refers to the superficially affected bone where only the cortical bone is necrotic, and soft tissue ulcerations are minimal. The therapy of this stage is mostly conservative. The second stage refers to the exposed cortical bone as well as smaller parts of the medullary bone. This stage is divided into group “a,” which includes minimal soft tissue ulceration, and group “b,” which refers to soft tissue necrosis and orocutaneous fistula. The therapy of this stage is mostly conservative or minor surgical intervention. The third stage clinically covers the entire thickness of the bone, and pathological fractures, fistulas, or necrosis of the surrounding skin are also possible. This stage is also divided into group “a” and group “b,” which refer to the same characteristics as in the second stage. This stage requires surgical intervention [51,52,55] (Figure 5 and Figure 6).

#### 2.2.4. Prevention

The first step in the prevention of osteoradionecrosis is a clinical examination of the patient before radiation. It is necessary to identify the teeth that need to be extracted, to inform patients about complications during radiotherapy and how to mitigate them, and to inform patients about the importance of preserving the health of the oral cavity and teeth after radiation [55,56].

Extraction should be performed before radiation in order to reduce the need for extraction during therapy and after radiation. It is necessary to extract all teeth with poor and questionable prognoses [52,57]. Extractions should be performed with as little trauma as possible with primary wound closure. Antibiotics are prescribed if signs of infection develop. Extraction should be performed at least 14 days before irradiation to allow sufficient time for tissue healing [11,58,59]. Extraction during radiation is not recommended as it may lead to interruption of treatment. Furthermore, the procedure will be difficult to perform due to mucositis [60].

Prevention continues even after the end of radiation treatment. The aim of prevention is to minimize the need for extraction, which is primarily done by intensive topical fluoridation of the teeth. Patients should be monitored regularly, at least every three months, for the cleaning of supragingival and subgingival deposits and reminding again of the importance of maintaining good oral hygiene [11,57]. If the extraction cannot be avoided, antibiotic prophylaxis should be used [57,61,62]. The most commonly used combinations of antibiotics are amoxicillin 500 mg and metronidazole 400 mg (every eight hours) or clindamycin 300 mg (every six hours). Antibiotic therapy should be started 24 h before the extraction and continue for five days after the extraction. The extraction should be performed with minimal trauma, and the wound should be mostly closed with sutures. The patient should be monitored until the extraction wound is completely closed [58]. Anesthetics without vasoconstrictors are recommended because some studies have shown an increased incidence of tissue necrosis if vasoconstrictors are used [57,63].

Hyperbaric oxygenation (HBO) has been used to prevent or treat osteoradionecrosis [11,55,64]. HBO therapy stimulates angiogenesis, induces neovascularization, optimizes oxygen content in osteoblasts, stimulates fibroblast and collagen production, and stimulates blood vessel ingrowth, which allows the treated bone to recover [64,65]. HBO therapy should not be applied in patients with chronic obstructive pulmonary disease, heart failure, and poorly controlled underlying disease [3]. Due to limited effectiveness and availability, high cost, and complications, it is rarely used as a therapeutic option [46]. Also, its effectiveness is debated in the literature [66].

Intensity-modulated radiation therapy (IMRT) allows the tumor to be irradiated with higher radiation doses and minimizes damage to adjacent normal tissue. By reducing radiation, it enables lowering the risk of osteoradionecrosis. It was shown that IMRT reduced the total maximum radiation and also reduced the volume of the mandible that was exposed to a higher dose of 50, 55, and 60 Gy. In addition, reduced exposure of the parotid gland to radiation resulted in improved salivary flow in the salivary glands and reduced xerostomia, thereby reducing the potential for caries and the need for tooth extraction. Thanks to targeted dosing, IMRT therapy consequently leads to a lower incidence of osteoradionecrosis [51].

#### 2.2.5. Risk Assessment

Patients with head and neck cancer who have received therapeutic radiation have a 2% risk of developing osteoradionecrosis. Certain factors increase this risk, such as invasive dental procedures (mostly tooth extraction), tumor localization, area of irradiated bone, oral hygiene, periodontal health, smoking and alcohol consumption, and uncontrolled diabetes [67]. The risk of developing osteoradionecrosis after a tooth extraction is 7% [48,68,69], and it is gradually increasing, reaching a peak five years after radiation [48,70]. For dental extractions between the second and the fifth postradiation years, the risk is 22.60%, and after that, it decreases to 16.7% [48]. It seems that the first postradiation year carries the lowest risk, probably due to partially preserved blood circulation in the bone tissue, but there is no consensus in the literature about the safest time interval for postradiation extractions. Wang et al. [70] suggest avoiding teeth extractions in the first four years after the radiation, which could be possible with good preventive procedures.

#### 2.2.6. Therapy

Treatment of osteoradionecrosis depends on the stage [52]. Conservative and surgical treatments are used in therapy. Conservative treatment comprises improved oral hygiene measures, use of antibiotics and analgesics alone or in combination with other methods such as ultrasound, hyperbaric oxygenation, and treatment with anti-radiation fibrosis drugs [3]. Surgical management includes different surgical methods of removal of dead bone and mucosa [51,52]. Stage I is treated conservatively with the addition of local antiseptic agents such as hydrogen peroxide and/or chlorhexidine. Stage II is treated with antibiotics and ambulatory wound cleaning. All necrotic bone is removed down to healthy tissue, and the wound is closed with sutures or soft tissue flaps. The third stage is treated by excision of the affected segment and reconstruction with free musculoskeletal flaps. For patients in the first and second stages, hyperbaric oxygen therapy is also recommended, with 20 treatments preoperatively and ten postoperatively [71], although available results from the literature do not support its use [63]. Conservative treatment consists of avoiding irritants such as tobacco and alcohol and adjusting dentures. Systemic antibiotics are used in acute infections, and if necessary, analgesics and anti-inflammatory drugs are also prescribed [11,51].

Ultrasound is used as a conservative treatment for osteoradionecrosis but also as an alternative for hyperbaric oxygenation. Ultrasound has been shown to increase angiogenesis and stimulate the formation of new collagen and bone. A treatment duration of 40 to 50 ten-minute sessions is suggested. Ultrasound can also be applied as prophylaxis before post-radiation tooth extractions [51].

Recently, successful cases of treatment of osteoradionecrosis with a combination of pentoxifylline with tocopherol, with or without clodronate, have been described. Pentoxifylline spreads blood vessels, reduces platelet aggregation, and inhibits inflammation; tocopherol or vitamin E has antioxidant properties, and clodronate acts by inhibiting macrophages and increasing bone formation [52]. Recently published review on this topic has shown that this type of treatment has promising results, but its effectiveness should be verified in controlled conditions with a larger number of patients [51,64,67].

Indications for surgical therapy include the third stage of the disease, involvement of the lower borders of the mandible, pathological bone fracture, and unsuccessful conservative treatment. The procedure includes resection of all affected necrotic parts of the bone, but also soft tissues, and primary reconstruction. Reconstructive methods include a bone flap or osteocutaneous microvascular free flap from the fibula, scapula, or iliac crest. No matter where the flap is taken from, the goals of mandibular reconstruction are to restore the lower third of the face and restore the patient’s ability to feed, speak, and breathe [51,55].

### 2.3. Other Causes of Osteonecrosis

The two most prevalent factors that favor the emergence of osteonecrosis are drugs or radiation treatment. However, osteonecrosis of the jaw occurs due to a variety of other factors, which are usually described in the literature only as case reports or case series. The causes can be divided into traumatic, non-traumatic, and idiopathic (spontaneous) [4,72]. Traumatic osteonecrosis is caused by thermal, mechanical, or chemical damage, while non-traumatic osteonecrosis is caused by neoplasms, infection, acquired and congenital diseases, and the use of narcotics [4,72]. Rare cases of idiopathic osteonecrosis have also been described [4,5].

#### 2.3.1. Trauma

Impact trauma is a frequent traumatic injury of the maxillofacial region, and it is most commonly caused by traffic accidents, violence, falls, and sports injuries [73,74]. Impaired arterial supply after traumatic injuries or after osteotomies may sometimes result in bone necrosis [72,75]. Suggested treatment consists of local debridement, antibiotic therapy, hyperbaric oxygen, and surgical reconstruction, if needed [72]. Placement or removal of an endotracheal or orogastric tube during anesthetic procedures or laryngoscopy rarely causes osteonecrosis of the jaw, but when it does happen, the tissue above the mylohyoid crest is most often affected. Conservative or surgical treatment is recommended [76,77].

Some dental materials, if used inappropriately, can cause osteonecrosis. Arsenic trioxide and formaldehyde-based materials serve as pulp devitalizing agents, but if these materials reach beyond the root canal system, osteonecrosis can occur [78,79,80]. These agents are not used today, but a root canal irrigant, such as sodium hypochlorite, if injected instead of a local anesthetic, can cause bone necrosis [81,82]. Acid etching during making fillings in rare cases can trigger gingival and bone necrosis, which can be treated surgically, even with a subepithelial connective tissue graft, if needed [83].

#### 2.3.2. Infection

Osteomyelitis is inflammation that affects the bone and manifests with discharging of pus from a wound, chronic abscess, development of fistula, and separation of devitalized bone. The exposed necrotic bone is not a typical feature of osteomyelitis [84].

Destruction of large areas of the soft and hard tissues of the head and neck can be seen in a gangrenous bacterial infection that is very rare today, noma (cancrum oris). The result are severe deformities of the affected areas. Predisposing factors are malnutrition, the impaired immune system of the host, and previous viral infection in combination with poor oral hygiene. Treatment includes blood transfusion, a high-protein diet, and antibiotic therapy [85].

Tuberculosis, syphilis, and actinomycosis can also, in rare cases, cause exposure, and necrosis of the bone can occur during infection, and the treatment consists of antibiotics, while in one case, a sequestrectomy was also required [86,87,88,89].

One of the causes of osteonecrosis can be infection with the herpes zoster virus. The pathophysiology of this type of osteonecrosis is not fully known, but various theories have been put forward. Some authors assume that blood vessels affected by a virus spreading from the cranial nerves show granulomatous vasculitis. Others report compression of the alveolar artery within its narrow bony canal by infection-induced nerve edema, leading to ischemia and then necrosis of the area. Clinically, this type of osteonecrosis is shown by exposed necrotic bone that was previously covered by ulcerated soft tissue, with classic herpes zoster symptoms such as vesicles on the innervation area of the affected branch and anesthesia of that area [90].

Fungal infections, which spread by inhaling spores, such as mucormycosis and aspergillosis, penetrate the blood vessels resulting in thrombi that lead to bone necrosis. Immunocompromised status, especially diabetes, is a predisposing factor. Aggressive surgery and rapid amphotericin B therapy are indicated as the most acceptable therapy [91,92].

The pulpal-periodontal process is an uncommon cause of osteonecrosis, and recommended treatment consists of tooth extraction and surgical removal of necrotic bone [93].

#### 2.3.3. Acquired and Congenital Diseases

Poor glycemic control in patients with diabetes mellitus results in microvascular changes increased susceptibility to infections and delayed healing. In these patients, denture trauma may result in necrosis of the jaw, as reported in the literature [94].

Different conditions inducing the development of microvascular thrombi and clot formation, such as disseminated intravascular coagulation (DIC), can rarely result in jaw necrosis [95].

Analogously, thrombophilia and hypofibrinolysis are also associated with the development of osteonecrosis. Thrombophilia is an abnormality in blood coagulation where the body tends to form blood clots, and hypofibrinolysis is a reduced capacity to break down fibrin in blood clots. Both of these conditions are associated with causing osteonecrosis since they disrupt the vascular supply of bone, leading to impairments in blood supply, ischemia, and ischemic bone death [96].

#### 2.3.4. Neoplasms

The metastatic spread of different tumors in the oral cavity can have various manifestations, including pain, inflammation, paresthesia, or necrotic appearance [97]. It is mandatory to exclude oral metastatic lesions before establishing a diagnosis of MRONJ, according to the definition of the American Association of Oral and Maxillofacial Surgery [6].

#### 2.3.5. Narcotics

The use of some narcotics can also result in bone exposure. Intranasal administration of cocaine, a narcotic drug that causes addiction, can lead to the development of oronasal fistula, palatal perforation, and soft tissue destruction. Administration of cocaine directly into the soft tissue can lead to gingival necrosis and bone exposure, which can cause small areas of necrosis and sequestration. Treatment consists of initial antibiotic therapy and removal of the necrotic area. Sometimes prosthetic rehabilitation is necessary [98]. Desomorphine, also called krokodil, is a cheap substitute for heroin. It causes necrosis of the midface, which requires surgical treatment [99].

#### 2.3.6. Spontaneous Osteonecrosis

In the literature, exposed bone in the maxillofacial region can be reported under various names, such as “oral ulceration with bone sequestration” [100,101]. It is a rare entity, and the literature data are scarce. Some described cases suspect lingual exostoses or prominent bone structures are potential predisposing factors [5,101,102]. Treatment options include conservative or surgical approaches [100].

## 3. Discussion

The incidence of osteonecrosis of the jaw is on the rise, primarily due to the increase of MRONJ and osteoradionecrosis. Besides these, a variety of possible causes should be taken into consideration. Osteonecrosis of the jaw is a diagnosis that is hard to cure, therefore, prevention should be our primary goal. Doctors of dental medicine and doctors of medicine should be educated about the known risk factors and give detailed instructions to the patient. If the patient is already under treatment, he should be sent to an oral surgeon or an oral medicine specialist. Specialists in oncology, hematology, and endocrinology should also be informed to refer patients to their doctors for dental medicine before they start their treatment. In Croatia, the doctor of dental medicine is not a member of the oncologic team, so the patients often come too late when the extraction is already needed and when it is too late for prevention. Patients’ history can give insight into drugs they are taking, which can sometimes allow us to foresee preventive procedures which could be made to motivate the patients to frequent check-ups, explain the importance of good oral hygiene, as well as topical fluoridation to preserve remaining teeth.

## 4. Conclusions

There is no unanimous protocol for the treatment of osteonecrosis of the jaw. The goal of the treatment is to relieve pain, eliminate infection, and slow or prevent further progression. More and more new drugs are coming to the market, the use of which can lead to the development of MRONJ.

Osteonecrosis of the jaw can be prevented by appropriate education of the doctors of dental medicine, doctors of medicine, and the patients themselves. As oral surgery poses the greatest risk for the development of osteonecrosis, doctors of dental medicine should be aware of the guidelines for patients undergoing radiation therapy or taking some type of antiresorptive or antiangiogenic therapy.

## Figures and Tables

**Table 1:** Types of osteonecrosis of the jaw.

Type	Cause
Medication related osteonecrosis of the jaw	Antiresorptive (bisphosphonates and denosumab) and antiangiogenic drugs (antineoplastics, e.g., bevacizumab, sunitinib); treatment with corticosteroids presents additional risk
Osteoradionecrosis	Radiation treatment in patients with head and neck cancer
Traumatic osteonecrosis	Thermal, mechanical, or chemical trauma
Non-traumatic osteonecrosis	Infection; malignancy; acquired and congenital diseases; use of narcotics
Spontaneous	Idiopathic

## Data Availability

Not applicable.
